# Theodicy and End-of-Life Care

**DOI:** 10.1080/15524256.2013.794056

**Published:** 2013-06-18

**Authors:** Simon Dein, John Swinton, Syed Qamar Abbas

**Affiliations:** Centre for Behavioural and Social Sciences in Medicine, University College London, London, United Kingdom; School of Divinity, History and Philosophy, King's College, University of Aberdeen, Aberdeen, United Kingdom; Palliative Medicine, St. Clare Hospice, Hastingwood, Essex, United Kingdom

**Keywords:** Christianity, end of life, God, Islam, Judaism, religion, suffering, theodicy

## Abstract

This article examines theodicy—the vindication of God's goodness and justice in the face of the existence of evil from the perspectives of Judaism, Christianity, and Islam. We focus on the thought processes that chaplains, social workers, and other professionals may use in their care interventions to address issues of theodicy for patients. Theodical issues may cause anxiety and distress for believers, but they can also potentially be a source of relief and release. Palliative care patients with a religious worldview often struggle with whether God cares about, or has sent, their pain. How social workers and other clinicians respond to such questions will have a great impact on how patients express themselves and use their religious beliefs to cope with their situations. For patients holding religious/spiritual perspectives, discussion of theodicy may facilitate closer relationships between patients and their caregivers and result in more compassionate and empathic care.

AB is a 60-year-old man with advanced pancreatic cancer whom you have been asked to see in a local hospice. Having been diagnosed 1 year previously, he now suffers with excruciating back pain and lethargy and has a prognosis of several weeks. He is receiving large doses of opiates. Labeling himself as religious, he regularly attended (church, synagogue, mosque) prior to becoming ill. During one consultation with a clinician he asks why God has allowed this to happen to him and admits that he is angry with God and feels deserted. He asks “Where is God now?” What should the response be?

## INTRODUCTION: SPIRITUALITY AND PALLIATIVE CARE

Spirituality is an integral part of palliative care. There is a growing health care literature that seeks to define the terms spirituality and religion and provide recommendations regarding spiritual care. Puchalski et al. (2009), in their recommendations from a consensus conference, note that studies have raised critical issues including the need for a commonly accepted definition of spirituality, the appropriate application of spiritual care in palliative care settings, clarification about who should deliver spiritual care, the role of health care providers in spiritual care, and ways to increase scientific rigor surrounding spirituality and spiritual care research and practice. As these authors point out, no one clinician can possibly meet the combined physical, psychosocial, spiritual, and personal needs of patients. While all team members have some responsibility for spiritual care, board-certified chaplains play a key role as the team member most directly responsible for this aspect of care and can play an important educational role in this respect with other team members. All members of the palliative care team should be trained in spiritual care at a level commensurate with their scope of practice in regard to the spiritual care model. These authors suggest a number of relevant topics including spiritual history taking and a knowledge of the options available for addressing patients’ spirituality—including spiritual resources, information, and indications for referral.

Although this journal is geared toward social workers, much of what we say is relevant to all health care professionals involved in end-of-life care. Most social workers would refer patients with spiritual issues to chaplains and rarely offer pastoral care interventions themselves. The term pastoral care refers to the ministry of care and counseling provided by pastors, chaplains, and other religious leaders to members of their church or congregation. But since they regularly encounter patients grappling with suffering, it is important, however, for them to understand how their chaplain colleagues deal with such issues.

This article will focus on exploring the potential implications of one particularly powerful set of Judaeo-Christian beliefs which have significant theoretical, pastoral, and clinical significance: the questions of *theodicy—*a vindication of God's goodness and justice in the face of the existence of evil. After outlining Jewish, Christian, and Islamic perspectives on suffering and their implications for pastoral care, we discuss how health professionals can deal with theodical issues in clinical care and describe how issues relating to theodicy can be incorporated into psychotherapy. The object of the article is to indicate the importance of recognizing the implications of specific religious beliefs for end-of-life care. It is not enough simply to know that a person is a Jew, a Christian, or a Muslim. It is the particular ways in which specific beliefs are perceived and worked out that contributes to health or ill-health. Here we examine the thought processes that chaplains, social workers, and other professionals may understand and use in their care interventions to address issues of theodicy for patients.

We focus on the monotheistic religions. Hinduism and Buddhism have their own frameworks for understanding suffering (for which see Peteet & D'Ambra, 2011). Some patients may not endorse any religious affiliation and still label themselves as “spiritual” whereas others might not profess any spiritual orientation or claim to be agnostics or atheists. For most patients, life threatening illness raises profound questions of meaning of their suffering; why has this happened, why am I suffering, and what will happen to me after I die? The emphasis of the article is on religion rather than on generic spirituality because this is where the bulk of the empirical research has been conducted. While religion describes the social, the public, and the organized means by which people relate the sacred and the divine, spirituality refers to such relations when they occur in private, personally, and even in eclectic ways. Although the two overlap, it is possible to be spiritual but not religious whereas the reverse is unlikely to be the case.

## RELIGIOUS STRUGGLES

By now there is a large literature suggesting that, on balance, being religious positively impacts upon mental health. Koenig, McCullough, and Larson's (2001) *Handbook of Religion and Health* presents several thousand studies which support this association. There is very little literature to suggest that generic non-religious spirituality is good for your health, mainly because there are so many different definitions that it is difficult to know what is being measured.

One area which has developed in the past decade is religious coping which is defined as “the use of cognitive and behavioral techniques, in the face of stressful life events, that arise out of one's religion or spirituality” (Tix & Fraser, 1998, p. 411). There is evidence that the particular ways in which people frame and explain their illnesses can have a significant effect on their ability to cope with their experiences. Spirituality and religion are common strategies for coping with life-threatening illnesses (Thune-Boyle, Stygall, Keshtgar, & Newman, 2006). Pargament (1997) asserts that religious coping serves a variety of functions such as finding and giving meaning, significance, comfort, belonging, problem solving, and spiritual orientation.

Pargament, Smith, Koenig, and Perez (1998) distinguish between positive and negative religious coping. The former reflects a constructive turning to religion for support, meaning and solace, and is generally adaptive, and is associated with positive mental health outcomes. The latter, in contrast, pertains to religious struggle and doubt and generally results in poorer psychosocial functioning. The two strategies are not mutually exclusive; some work in oncology suggests that patients predominantly rely on positive religious coping and only deploy negative religious coping to a limited degree (Sherman, Simonton, Latif, Spohn, & Tricot, 2005).

Exline and Rose (2005) reviewed four types of religious struggle leading to negative coping: (a) suffering (i.e., blaming God for any suffering and being angry at God); (b) virtuous striving (i.e., blaming and not forgiving the self if the person falls short of the virtues cultivated within the religion); (c) perception of supernatural evil (i.e., blaming evil forces or believing in possession by a diabolical force); and (d) social strain (i.e., feeling hurt or offended by the religious community or feeling like an outcast by a religious group). Studies suggest that negative religious coping is associated with more psychological distress and increased mortality risk (Exline, Yali, & Lobel, 1999). We now focus on one type of religious struggle—theodicy.

## THE QUESTION OF THEODICY

The term theodicy was introduced into philosophy by Leibniz, who, in 1710, published a work entitled: *Essais de Théodicée sur la bonte de Dieu, la liberté de l'homme et l'origine du mal* (*Essays of Theodicy on the Goodness of God, the Freedom of Man and the Origin of Evil*), often shortened to *Théodicée*. The book was written after the horrors of the Lisbon earthquake. However, while Leibniz introduced the term theodicy, the issues that he wrestled with are much older. David Hume traces the fundamental question of theodicy back to the philosopher Epicurus: “Epicurus’ old questions are yet unanswered. Is he [God] willing to prevent evil, but not able? Then he is impotent. Is he able, but not willing? Then he is malevolent. Is he both able and willing? Whence then is evil?” (Hume, 1980, p. 198). Translated into a clinical context: “How could a God of power and love allow this to happen to me?”

Suffering raises profound questions about the role of God in human affairs and for believers can create a state of cognitive dissonance. Religious believers seek to answer such questions by turning to their traditions. So, for example, the person might attribute only goodness to God in which case the suffering may be attributed to other causes such as evil, sin, or something that the person feels he/she has done wrong to deserve such punishment. Similarly, the illness might be attributed to a lack of faith or a testing of their faith, original sin; atonement for individual sin; character building; the result of free will, illusory; God's unknown purposes; educational and increasing faith and so forth. The key point is that the person's religious tradition provides the interpretative lens through which is worked out personal theodicy. This may sit within established theodical understandings as laid down by religious traditions or it may sit outside of such theodicies. For the practitioner, the issue is to ensure that she has enough knowledge of the particularities of theodicy to enable her to recognize and support the wrestling of the patient. Through a deeper understanding of the particular meaning of patients’ theodical beliefs, empathy, compassion, and understanding will be increased and patient care enhanced at a fundamental level.

Put simply, *theodicy is the intellectual defense of God in the face of evil and suffering*. Theodicies are designed to provide explanations for evil and to enable people to hold on to the possibility of God in the midst of pain and suffering and seek to provide complex philosophical and theological arguments to justify and sustain the idea that there is logic in believing in a God who is perfectly good, all loving, and an all powerful God, even in the face of the reality of the world's pain. Two influential Christian theodical arguments that have been deeply influential in shaping how Western religious traditions have come to understand evil and suffering come from the theologians Augustine and Irenaeus.

### Augustinian Theodicy

The philosopher John Hick (1966), in his book *Evil and the God of Love*, lays out two ways of explaining evil and suffering which he draws from St. Augustine and St. Irenaeus. Augustine viewed evil, not as a thing in and of itself, but as a deprivation of the good creation of God. God is all good and all that God creates is necessarily good, so the presence of evil could not be caused by God in a direct fashion. So how could evil and suffering exist? As God is not capable of creating that which is evil, evil cannot be an entity, a “thing” with substance and purpose. Rather, it is a deprivation of the good; what might be described as a hole in the goodness of God's creation. Evil does not have a positive nature. It is nothing more than the loss of the good. As all goodness comes from God, evil is ultimately a turning away from God. How then is the goodness of creation lost? Augustine finds the answer in human free will. Humans were given free will and allowed to choose for God or not for God. They chose the latter and evil, sin, and suffering are the consequences of that decision. While moral evil is not unconnected with the experience of terminal illness, it is natural evil that forms the core of medical theodicy: “Why me, why now, and why this illness?”

### Irenaean Theodicy

Third-century philosopher and theologian Irenaeus offers a different theodicy. Within his understanding, suffering has a more positive role and persona. God creates a world within which suffering is possible because only in such a world could we learn to love and to care. In order that we are not overwhelmed by God's love and beauty, it is veiled by the tragedies of this world. If we did not have evils to veil God's irresistible beauty, it would not be possible for human beings freely to choose to love God. (i.e., God would be irresistible). In this theodicy, life is viewed as a journey within which one gradually gains knowledge and love of God as one encounters life's trials. Evil and suffering provide human beings with the necessary challenges and problems through which they are enabled to participate in what Hick (1966) calls “soul-making.”

It is easy to see how patients might work implicitly or explicitly with the Augustinian and Irenaean theodicies. Simper expressions such as “I guess it's my own fault” or “It's all for the good” indicate implicit or explicit adherence to either or both of these theodical structures. Importantly, both of these theodicies are Christian and both say quite different things. That being so, two patients from the same religious tradition could be working with very different interpretations of the meanings of their illness. It's not enough just to know a person's general faith orientation.

### Practical Theodicy

At an academic level, theodicies provide complex philosophical and theological arguments to justify and sustain the idea of a loving, all powerful God in the face of the human experience of pain and suffering. However, at a personal level they serve to provide a variety of powerful explanatory frameworks. This tension between the general and the personal is important. Recently, scholars and practitioners have begun to recognize that the questions that theodicy raises have *practical* as well as philosophical and theological importance (Swinton, 2007). Anecdotal reports from pastors and chaplains alongside the limited research that has been done on the pastoral implications of theodical beliefs indicate that the ways in which such questions are answered by individuals in times of suffering can significantly impact upon their mental health and well-being at the end of life. This, combined with observations from psychology that it is the particularities of religious belief that brings health and healing (see Pargament, 2002), makes theodicy a particularly relevant set of theological beliefs which can be identified and explored.

To date, the work examining theodicy in end-of-life care is limited. In a study of caregivers of terminally ill patients, Mickley, Pargament, Brant, and Hipp (1998) found that caregivers of patients with cancer who appraised their situation as part of God's plan or as a means of gaining strength or understanding from God reported positive outcomes while those who viewed their situation as unjust, as an unfair punishment from God, or desertion from God had low scores on mental and spiritual outcomes. Francoeur, Payne, Raveis, and Shim (2007) suggest that patients who believe suffering to be redemptive might also be receptive to trusted clergy and pastoral counselors from their faith tradition who encourage them to consider acceptance of caregiving from family members and health professionals. This may also be another means for acceptance of God's will and mercy, not only for the patient, but perhaps as well, or even more so, for other family members who are provided an opportunity to atone for their own sins by providing care and relieving suffering in a loved one. Thus there is evidence that theodical issues impact upon well-being at end of life.

The focus of this article is on the clinical implications of theodicy; what does theodicy actually look like in this practical mode? In what follows we will offer a brief overview of theodicy as it relates to three religious traditions: Judaism, Christianity, and Islam, and offer some potential pastoral insights that will help locate the theoretical discussion within clinical practice. Other world religions must also explain the presence of evil in the world, but the problem is particularly complex for Judeo-Christian traditions because of the assertion that God is both all powerful and all loving. We emphasize that although there is a potential diversity in interpretations of suffering possible within each religion, we cannot assume that all adherents of these three religions are likely to see the world in this way. Furthermore, the question of how to explain evil is not limited to the theistic religions. It can be equally challenging to explain evil in the context of evolutionary theory and other spiritual or faith-based traditions that do not believe in a theistic god. We shall begin with Judaism.

## THEODICY IN JUDAISM

Krell (2011) has provided an excellent overview of theodicy in Judaism and the discussion here derives from him. There are diverse responses to the problem of suffering and the role of God in this process and here we provide a brief overview. In the Biblical and First Temple periods there is no discussion of suffering and God is assumed to be just. Instead there was a sense of collective responsibility for evil as sin: suffering and sin run closely together. Both Leviticus and Deuteronomy advocate punishment for not heeding God's laws.

The Book of Job in the Second Temple period, influenced by Hellenistic thought, expresses a tension between an underlying faith in God's justice and a protest in which God is put on trial following Job's personal tragedy. This text rejects the view that suffering is always deserved punishment, and ultimately suffering is a mystery. At the end Job's complaints remain unanswered and he is blamed for being ignorant of God's Divine providence. Another view is that God actually brings suffering upon those he loves to relieve them of their sins and re-establish a purified relationship with him. “After he has suffered, he will see the light of life and be satisfied; by his knowledge my righteous servant will justify many, and he will bear their iniquities” (Isaiah 53:1). The fact that the righteous suffered in this world could be accounted for by the fact that they would be rewarded in the *olam ha bah* (the world to come), and receive joy and liberation. Such a view is expressed in the Medieval Kabbalistic text, the *Zohar* (BT Kiddushin 40b): “God gives pain to the righteous in this world in order to make the righteous meritorius for the *Olam Ha-bah*.”

Another popular, though controversial, approach to theodicy is that God is not all-powerful. Harold Kushner (1981), in *Why Bad Things Happen to Good People*, provides an in-depth description of theodicy in Judaism. For him God is not omnipotent in the classical sense and is less than perfect. He contends that bad things happen to good people because: (a) it sometimes just goes that way, God made the world with natural laws including those of cause and effect; (b) we are given freedom of choice and consequently life is full of injustices; (c) Nature is morally blind; and (d) there may be “corners” of the universe where God's creative light has not yet penetrated. According to Kushner, God is not ignoring your suffering when he doesn't act to prevent it because—as an all-knowing God—he is aware of your suffering. As a perfectly good God, he also feels your pain. The problem is that he can't do anything about it because he's not omnipotent.

To summarize, some of the main ways that the Jewish tradition perceives suffering and which may underlie the responses of patients facing death and suffering are as follows:

- Punishment: The affliction has been inflicted as a direct action of God.
- Nothing to do with God: Evil simply happens. It has nothing directly to do with God. Silence is the only appropriate response (anti-theodicy).
- Mystery: We do not know why suffering exists but what we do know is that God is present within it.
- God is neither omnipotent nor perfect. We therefore should not be surprised that suffering exists and we should not expect God to take us out of it.

Rabbi Miriam Klotz (2005) offers valuable insights for those working with suffering in a Jewish context. She asks how we can maintain or be open to a sense of God's presence during periods of inexplicable suffering. Who or what is a God who would allow such hardship? Within Jewish thought there is a paradoxical tension between certainty in the revealed presence of God in the world and the submission to a mysteriously elusive force which is beyond human comprehension; God is both known and unknowable, perhaps best exemplified liturgically in the *Kedusha* prayer recited on the Sabbath and festival morning services.

Klotz (2005) asserts that at times, gently encouraging sufferers to articulate the meaning or context of their suffering may be appropriate. This includes a discussion of God's role in suffering through theological reflection. This may ultimately result in increased resiliency. There are dangers in imposing interpretations on a person's suffering. Job makes the important point that providing explanations for suffering can heighten the alienation experienced. Rather, developing a caring relationship with sufferers in the presence of mystery can facilitate healing. It was not God's answers but his presence that healed Job. And Job himself was not concerned with discovering why he suffered, but only with feeling God's presence. Although it is not the job of the pastoral or other caregiver to “explain away” suffering, providing sufferers with theological perspectives involving God's role in suffering can act as a springboard for meaning. These perspectives need to be tailored to suit particular individuals going through unique situations and should not be approached as an academic discipline. At times individuals’ theological positions may be “pathological,” as in the case of excessive guilt—this may need to be gently challenged by social worker, chaplains, and others.

Although explanation may be important for some, Klotz (2005) makes the pertinent point that regardless as to *why* a person suffers it is important to provide an appropriate *response* to this suffering—a shift from “Why me?” to “How am I going to respond to this suffering?” Meaning can often be found in the response to suffering more than in understanding why it happened. In practice we cannot eradicate our powerlessness and helplessness over the powers of life and death that are present in the world and people suffer in a random way. We can change the way in which we respond and in turn facilitate a sense of choice. Specific Jewish responses include repentance (*teshuvah*)— turning away from past mistakes, prayer, and deeds of righteousness.

## THEODICY IN CHRISTIANITY

Sulmasy (1999) offers a fourfold structure to describe what we might describe as classical Christian theodicies.

### Privation Theories

Here we encounter the theories of thinkers such as Augustine, Aquinas, and Leibniz. Within this perspective evil is conceived as a deprivation of the good rather than an entity unto itself. Evil does not exist in an ontological sense; it is simply a deprivation of the good brought about by human free will and human disobedience. It will eventually be overcome as creation is realigned with God. Within this perspective the incarnation (God becoming human in Jesus) and Jesus’ redemptive mission and death are central insofar as they are indicators that God's goodness continues to reign and that through the death of Jesus the consequences of human disobedience have been overcome. In this way a hopeful future becomes possible and available for those who choose to be with God.

### Proving-Ground Theories

Under this heading comes the type of Irenaean theodicy we discussed previously. Overcoming suffering and evil is understood as a way of moving toward God. Once again evil and suffering don't really exist. Things certainly feel evil and suffering certainly is a problem; however, both evil and suffering are actually ways of accessing the ultimate good and in the long term the essential goodness of the universe is sustained.

### Process Theology Approaches

Within the process perspective, how a God of love and power can allow evil is overcome by proposing that God is not in fact all-powerful; God is limited (Cobb, 1969) God does not control the future in the way that a strict view of providence might suggest. God therefore neither causes, nor removes evil. He simply walks with the sufferer in history toward an endpoint that has no guarantees. He has no power to end evil or suffering other than the power of persuasive love. God is the indirect creator of evil in that he has persuaded creation to bring forth entities that have the potential for evil. However, as God always intends the good, he is not blameworthy and always shares in the suffering within a creation that is both beautiful and tragic (Migliore, 1991; Farley, 1990).

### Existentialist Approaches

These approaches begin with the premise that evil cannot be explained; that it is a mystery (Surin, 1986). To ask for an explanation is simply to reveal one's post-enlightenment rationalistic roots and one's inability to live with unanswered questions. Modernity has a tendency to turn mysteries into puzzles, but when it comes to suffering and evil there are no answers. The problem of evil and suffering is not a puzzle to be solved but an experience to be lived with. To summarize, some of the main ways that the Christian tradition perceives suffering and which may underlie the responses of patients facing death and suffering are: privation theories; proving-ground theories; process theology approaches, and existentialist approaches.

We can see how each one of these theodicies could lead to a different framing of terminal illness which in turn would require a different response. If Pargament (2002) is correct in emphasizing the particularities of religious beliefs over and against our global assumptions, then this is no small point.

### Privation Theories

Patients might come to a position wherein they believe that their illness is the product of sin, or/and that their suffering is within God's providential plan. This could be positive or negative. For example, positively, it could provide an explanation that brings the situation under control. Even in the midst of the confusion of illness and suffering, God is guiding the situation toward a hopeful conclusion. Negatively, sufferers could come to associate their illness with particular sins (as opposed to general, original sin). If this happens, issues of guilt, hopelessness, and alienation from God become significant.

### Proving-Ground Theories

Within this framework, patients might be encouraged to work with their illness in positive ways, perceiving it as an opportunity to come to know God more deeply and perhaps come to know themselves and others better through this process. Negatively, patients might frame their illness in overly positive ways which prevent positive intervention, and/or develop fatalistic rather than proactive responses to their suffering. The key thing to observe in this theodicy is the underlying assumption that God is good, that life has meaning, and that God is guiding a person through their suffering toward a meaningful and hopeful end point.

### Process Theology Approaches

Those patients who might hold some derivation of the process approach might simply feel that their suffering is just the way things are. God is close to them but they realize that his intervention will be minimal. People within this theodical perspective might perceive medicine as their primary source of healing with God as simply an accompanying sufferer rather than a proactive intervening agent. So the person might be deeply religious, but not expect from God the kinds of things that someone with a strong doctrine of providence and an expectation of an interventionist God might expect.

### Existentialist Approaches

Patients with this perspective may perceive their illness differently from those who adhere to the other frameworks. They will not tend to try and spiritualize away the pain of suffering. For these patients the question will not be why do evil and suffering exist, something we cannot know at present, but rather what do evil and suffering *do*? How does it impact upon my faith and how can I hold onto my faith even in the midst of this suffering. Here the redemptive sufferings of Jesus have particular significance. On the cross Jesus died with and for human beings. Jesus remains in solidarity with those who suffer. Suffering remains mysterious, but it is not without hope. In this way, the theoretical questions are reframed as practical ones. Faith must be sustained in the midst of suffering and unanswered questions. Patients working out theodicies from this perspective will be focused on how they can retain their relationships with God even in the midst of their suffering. Prayer, meditation, and scripture reading are all perceived as modes of resistance, ways of reinforcing the redeeming presence of God even when things look appear to be quite the opposite.

## THEODICY IN ISLAM

From the very beginning of Islamic preaching in the early 7th century, there were questions about theodicy. Islam has adopted a number of frameworks to account for suffering which we discuss below.

### Achieving Self-Realization in the Face of Suffering

According to this perspective suffering is a creation of God; however, it is not necessarily brought by God, but is rather a means to achieve realization of the self. Ibn Sina (980–1037 AD) maintains that the highest form of pleasure is to seek and reach the First or Essence (i.e., God). However, in order to reach this state, we must be able to acquire this pleasure. One obstacle in acquiring this pleasure is its lack of appreciation. He gives the example of a healthy person who may not appreciate his condition of health unless he becomes sick himself. His sickness allows him to appreciate the condition of being healthy and so now he is both *intellectually aware* of what it means to be healthy and has *fully acquired* health. Taking the lead from this, perhaps we can infer that suffering is a necessity in our journey to God because by gaining self-awareness of our circumstances, we can better appreciate real happiness—God (Inati, 1996).

### Suffering Containing Inherent Goodness

In the same century, Persian theologian and philosopher Al-Ghazali (1058–1111) continued this topic and maintained his interest in his book *Al-Maqsad Al-Asna Fi Sharh Asma’ Allah Al-Husna* (*Ninety-Nine Beautiful Names of God*). He states that if God is so merciful, why doesn't he remove human afflictions? His answer is that:

All existing evil has some good in it. If that evil is removed, surely the good inherent within it will become ineffectual. Subsequently by means of the nullity of the evil itself, an even greater evil results. (Al-Ghazali, 1970, p. 16)

He gives the following example to illustrate his point:

Since this is the case, even though the amputation of the leprous hand appears to be an evil, inherent in this act is ample good, namely, the well-being of the total body. Furthermore, if the amputation of the hand is omitted, the destruction of the entire body would ensue, and then (certainly) the (ultimate) evil would be greater. (Al-Ghazali, 1970, p. 16)

### Looking Beyond the Idea That Suffering Is Something Only Related to This Life

Whatever God intends through His infinite mercy is not merely for the life of this world but for the ultimate happiness of the next. Hence, suffering should not merely be viewed as a negative condition that permanently leads to unhappiness in this life. Rather, it is linked to the ultimate happiness of the next life and as Al-Ghazali (1970) states later, contains an inherent goodness within it.

### Suffering as a Means for Returning to God

This discussion was carried forward by the early 17th century's Persian scholar Mulla Sadra (1571–1641). He holds that each soul originates with God and one's journey is completed only when the soul has returned to Him. Commenting on the wisdom behind the creation of Satan in Chapter 9 in *Iksir al-Arifin* [*The Elixir of the Gnostics*], he states that when a person becomes weary of the trials and tribulations of this world and is driven away from the world's creatures and happiness, his nature is to flee from them. But flee toward God and cling to the Causer of Causes and the Easer of affairs—“So flee unto God”. Perhaps this is relevant to suffering if we consider suffering as a means to reaching God—of giving a person the awareness that the happiness of this world is only temporary. It is here that a person realizes his love and need to cling toward God and reach the closeness and happiness that God is preparing Him for (Sadra, 2003).

Thus suffering can be seen as: an atonement for sin, a natural occurrence, a test which deepens faith and leads to self-actualization and a form of goodness which might only be manifest in the afterlife. Based on such background, the discussion about the suffering at the end of life leads to an understanding of moving to an eternal place, which is inevitable. There is a strong belief that all good and repenting souls will be rewarded. The suffering in this world leads inevitably to solace in the next. Islamic chaplains also discuss the spiritual means to ease such suffering like recitation of specific verses of the Quran and reflection on these. The verses commonly recited to achieve solace and peace before death are from 36th chapter, *Ya-Sin* (Alphabets).

## CLINICAL IMPLICATIONS

What are the implications of theodicy for health practitioners? Theodical issues may cause anxiety and distress for believers, but they can also potentially be a source of relief and release. Patients with a religious worldview often struggle with whether God cares about, or has sent, their pain. How clinicians respond to such questions will have a great impact on how patients express themselves and use their religious beliefs to cope with their situations. For patients holding religious/spiritual perspectives, discussion of theodicy may facilitate closer relationships between patients and their caregivers and result in more compassionate care. However, it is not only those who profess religious/spiritual beliefs who have to deal with suffering. Atheistic patients also search for the meaning in their lives but reject the answers offered by traditional authorities. It is interesting to note that within secular Britain, the phenomenon of anthropodicy—an attempt, or argument attempting, to justify the existence of humanity as good—is not uncommon: “What did I do for this to happen? Was it my diet? My lack of exercise? My mother's genes?” (Swinton, Bain, Ingram, & Heys, 2011). While our focus here has been on religious beliefs, the basic psychological dynamic is present in many different kinds of situations with patients with diverse beliefs.

The implications of theodicy are both contextual and multifaceted. Social workers and other health professionals should work together in sharing or respecting these theodical possibilities/perspectives with patients and families. This type of coordinated care could especially be important for Muslim patients and family members who may feel the need to refuse palliative care to relieve symptoms such as pain because they consider their suffering to be God's will. In other situations, patients may not feel comfortable with clergy or pastoral care counselors, especially patients who feel alienated from, or who no longer believe in, the faith tradition of their earlier life, as well as agnostic patients. Social workers may need to draw on theodicy perspectives from all three of these faith traditions, and perhaps other traditions, in order to help these patients (and family members) who may be “spiritual but not religious.” Sometimes health professionals may lack knowledge of working with these issues and referral to chaplains is appropriate in these situations. Indeed, some health professionals, including clinical social workers, might rightly reply that their “correct response” is to refer the patient to a spiritual care professional in recognition that their expertise is limited in this field.

There has been recent interest in the ways in which health professionals can work with theodical issues in therapy with patients with life-threatening illness. One of the most important reasons for therapists to have training in religious and spiritual issues is to avoid unintentionally imposing their values on their clients through misunderstanding or not being familiar with the client's belief system. It is important to be aware of the different approaches that individuals take to resolve these issues. While it is unrealistic for professionals to gain knowledge of all the issues that clients are dealing with, it is important to gain some awareness of theodicy especially if psychotherapeutic work with clients is contemplated. Although most social workers do not conduct psychotherapy with dying patients, preferring short-term work, some may choose to further train in this area.

Hoffman, Grimes, and Mitchell (2010) describe a psychological intervention for clients struggling with issues of theodicy and other forms of suffering and loss which involves working with images of God and facilitating patients’ expression of anger toward him. They utilize Ana-Maria Rizzuto's (1979) object relations framework for understanding representations of God. This framework differentiates between one's *concepts* of God and one's *images* of God. The God Concept, defined as a person's cognitive beliefs about God or a transcendent other, is contrasted with the God Image, a person's emotional or relational experience of God. Both can be powerful sources of sustenance and healing when they are internally consistent and healthy. However, when there are unresolved discrepancies between them, or when they entail negative experiences of God, these psychological processes often further complicate the healing and growth processes. Through therapy, individuals are helped to resolve these discrepancies. In this framework theodicies are seen as defenses which may facilitate coping but can also lead to greater suffering.

Although religious groups have traditionally discouraged the expression of anger toward God and other people, often associating anger with sin, in the Jewish and Christian scriptures anger is commonplace: God is angry, Jesus displays anger, and anger expressed toward God is commonplace among the prophets. This is to be contrasted with Islam where expression of anger to God is unacceptable. A critical and foundational issue in dealing with theodicy, evil, and suffering in therapy is the ability to be able to tolerate the client's anger. It is important to provide a safe space for patients to express their anger toward God and to question his actions. However, it is not the therapist's responsibility to find or create the answers. It is becoming increasingly common for therapists to embrace ideas such as mystery, questioning, and spiritual journeying as healthy spirituality (Moore, 2002; Schneider, 2004).

## CONCLUSION

It appears that theodical questions are commonplace at the end of life in those with life-threatening illness. Pastoral care in all three faith traditions emphasizes moving beyond the provision of appropriate intellectual responses to the provision of theologically appropriate practices including prayer and ritual. Dealing with theodical issues in the clinical context can facilitate closer caring relationships between health professions and their patients at the end of life and results in more compassionate care. To this extent it is necessary for those working in this context to have knowledge of theodicy.

The literature on religion and coping has evolved considerably since the publication of Pargament's (1997) *The Psychology of Religion and Coping: Theory, Research, Practice* to examine the specifics of religious coping. The area of theodicy has, however, been neglected. This is an important focus for future research. Questions might include the following: how common are theodic questions; how do sufferers and their caregivers attempt to answer them; and what impact do they have on mental health? Knowledge of theodicy will not only further the field of the role of religious and spiritual variables in coping, but also provide potential interventions which can be deployed in religious psychotherapy and in turn help provide more compassionate care for our patients.
